# Path-dependence of the Plio–Pleistocene glacial/interglacial cycles

**DOI:** 10.1073/pnas.2322926121

**Published:** 2024-06-17

**Authors:** Judit Carrillo, Michael E. Mann, Christopher J. Larson, Shannon Christiansen, Matteo Willeit, Andrey Ganopolski, Xueke Li, Jack G. Murphy

**Affiliations:** ^a^Department of Earth and Environmental Science, University of Pennsylvania, Philadelphia, PA 19104; ^b^Potsdam Institute for Climate Impact Research, Potsdam 14412, Germany

**Keywords:** climate models, atmospheric carbon dioxide, glacial-interglacial cycles, hysteresis

## Abstract

We find strong path dependence in the evolution of the Plio–Pleistocene glaciations using CLIMBER-2 Earth System Model simulations from the mid-Pliocene to modern preindustrial (3 My-0 My BP) driven by a gradual decrease in volcanic carbon dioxide outgassing and regolith removal from basal ice interaction. Path dependence and hysteresis are investigated by alternatively driving the model forward and backward in time. Initiating the model with preindustrial conditions and driving the model backward using time-reversed forcings, the increase in volcanic outgassing back-in-time (BIT) does not generate the high CO2 levels and relatively ice-free conditions of the late Pliocene seen in forward-in-time (FIT) simulations of the same model. This behavior appears to originate from nonlinearities and initial state dependence in the carbon cycle. A transition from low-amplitude sinusoidal obliquity (~41 ky) and precession (~23 ky) driven glacial/interglacial cycles to high-amplitude ~100 ky likely eccentricity-related sawtooth cycles seen between −1.25 My and −0.75 My BP (the Mid-Pleistocene transition or “MPT”) in FIT simulations disappears in BIT integrations depending on the details of how the regolith removal process is treated. A transition toward depleted regolith and lowered atmospheric CO2 levels are both required to reproduce the MPT.

## Significance

No paleoclimate phenomenon has been more vexing to the climate modeling community than the Plio–Pleistocene glacial/interglacial cycles. We demonstrate that by using time-reversed forcings, model simulations do not generate the high CO2 levels and relatively ice-free conditions of the late Pliocene, as seen in the forward-in-time simulations of the same model. We also find that a transition toward depleted regolith and lowered atmospheric CO2 levels is required to produce the mid-Pleistocene Transition (MTP) toward large-amplitude ~100 ky sawtooth glacial/interglacial cycles. We show that these features originate from nonlinearities and initial state dependence in carbon cycle behavior.

A critical test for climate models used to project future human-caused climate change is their ability to explain past natural changes. No paleoclimate phenomenon has been more vexing to the climate modeling community than the Plio–Pleistocene glacial/interglacial cycles. Explaining the Mid-Pleistocene Transition (MPT), the transition between 1.25 and 0.75 Mya from predominantly obliquity-paced (~41,000-y) to an ostensible eccentricity-paced (~100,000-y) cyclicity in climate state variables such as global ice volume, atmospheric CO_2_, and global surface temperature has been particularly challenging, given the absence of any substantial changes in the underlying insolation forcing (1–16). Prior to the MPT, insolation maxima were associated with deglaciation.

Changes in insolation forcing alone cannot explain the Plio–Pleistocene glacial rhythmicity including the ~100-ky glacial/interglacial cycle and its onset (2, 8, 11, 15, 17, 18). A number of hypotheses have instead been offered to explain the observed history. These include obliquity-period-doubling bifurcation as the result of increased amplitude feedbacks during the MPT (19), synchronization of internal self-sustained oscillations to 100-ky eccentricity cycles with the help of the 41 ky obliquity forcing (20), and nonlinear responses to eccentricity forcing (17). Other hypotheses involve changes in ocean carbon chemistry changes resulting with changing aridity (1), threshold-dependent feedbacks associated with ice sheet extent (2, 4, 15, 21), changing oceanic and atmospheric poleward heat flux (14, 18), changes in ocean stratification impacting ocean-atmosphere CO_2_ exchange (5), regolith erosion (6, 16, 17, 22, 23), carbon cycle dynamics (7, 12, 24), Atlantic meridional circulation changes (9), bedrock depression (10), and dust fertilization of the Southern Ocean (13).

Willeit et al. (23) recently used simulations of the past 3 My with the CLIMBER-2 Earth System Model of Intermediate Complexity to reproduce the main features of the Plio–Pleistocene glacial/interglacial cycles. The model, which includes coupled atmosphere, ocean, ice sheets, and carbon cycle components, was driven by Earth orbital forcing in addition to long-term tectonic forcing from carbon dioxide (CO_2_) volcanic outgassing (3).

Willeit et al. also included a key geological driver that had not been included in past modeling work. Ice flow by increasingly large Quaternary ice sheets led to substantial advection of unconsolidated sediment (regolith) from the center to the periphery of ice sheets, exposing the bedrock beneath, and reducing basal velocities by a factor as large as five, increasing ice sheet stability and reducing susceptibility to orbitally induced surface melt (2, 4). Regolith also modifies the surface albedo when deposited on the ice sheet surface, facilitating melting and further preventing the growth of large ice sheets (3). By accounting for the effect of regolith removal in the behavior of the North American and Eurasian ice sheets, Willeit et al. were able to reproduce not only the main characteristics of the Plio–Pleistocene glacial/interglacial cycles but also effectively captured the onset of the MPT (23). The removal of regolith and the consequent growth of larger ice sheets, in the simulations by Willeit et al., lead to the transition toward the approximate ~100,000-y period sawtooth glacial/interglacial cycles.

Because of the nonlinearities in the underlying physics and biogeochemistry, several Earth system components relevant to the Pleistocene glacial/interglacial cycles are capable of exhibiting hysteresis and path dependence, including ice sheet dynamics (25, 26), the ocean circulation (27), and carbon cycle processes (28, 29). In this work, we find that such path dependence appears to be intrinsic to the behavior of the coupled ocean–atmosphere–cryosphere system and the details of the glacial/interglacial cycles that characterize the Plio–Pleistocene interval. We investigate this path-dependence using the same (CLIMBER-2) Earth System Model used by Willeit et al. However, we alternatively drive the model both forward and back-in-time (BIT) with the same Earth orbital, tectonic, and geological forcings, testing the sensitivity of the behavior of the system to initial conditions and prior model states. We test alternative regolith configurations to assess the sensitivity of the MPT to the precise handling of regolith removal. We examine the feedbacks and nonlinearities associated with atmospheric CO_2_, ocean circulation, ice volume, and carbon cycle dynamics to assess the precise factors that govern the detailed behavior of the Plio–Pleistocene glacial/interglacial cycles.

The model employed in our experiments (CLIMBER-2), as any intermediate complexity model, requires a substantial simplification of many of the underlying oceanic, atmospheric, and biogeochemical processes relevant to long-term climate variability, and the results in some cases can be sensitive to the particular value of various certain model parameters. For example, modest changes in ocean vertical diffusivity in CLIMBER-2 can impact the stability of overturning ocean circulation (30). Because our simulations are based on this single model, and because long-term transient simulations of glacial/interglacial cycles like those of Willeit et al, while promising, are still in their relative infancy, the results are not a definitive characterization of climate system behavior. Rather, they should be thought of as providing evidence of dynamical behavior that is worthy of further investigation through multiple modeling frameworks. That is the spirit in which this investigation is undertaken.

## Results

### Model Simulations Results.

#### Confirmation of previous findings.

**Fig. 1.**
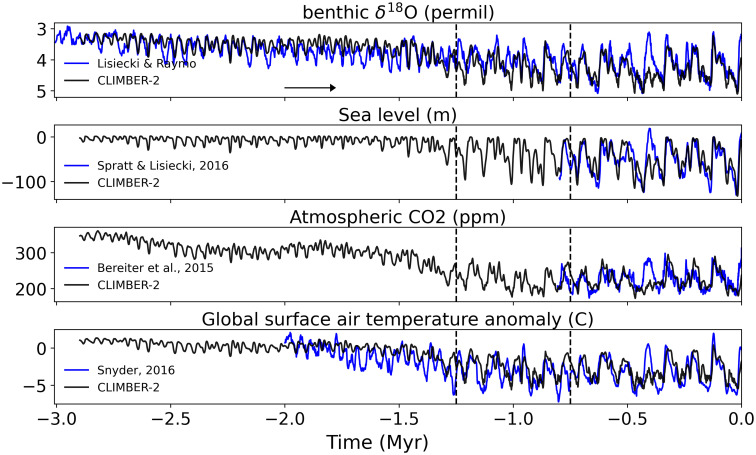
Standard (continuous) simulation from −3 My to present. Modeled benthic δ^18^O, relative sea level, atmospheric CO_2_, and global surface air temperature (all relative to modern preindustrial) (black) compared to the proxy estimates of these quantities [respectively from Lisiecki and Raymo (31), Spratt and Lisiecki (32), Bereiter et al. (33), and Snyder (34)] (blue). The arrow indicates the direction of execution of the simulation. The dashed vertical lines mark the MPT. The simulation is driven by orbital forcing, optimal regolith removal scenario (initial and final spatial distributions of regolith are limited by empirical data), and prescribed long-term decline of volcanic CO_2_ outgassing (*SI Appendix*, Fig. S1). This simulation constitutes our standard FIT simulation.

We first reproduced the basic findings of Willeit et al. (23) using a simpler, continuous-execution approach over the full 3 My Plio–Pleistocene interval (the original authors used a time-splitting technique to optimally reproduce each of a number of distinct time segments). Fig. 1 compares the results of our standard [forward-in-time (FIT)] experiment with proxy measures of ice volume (δ^18^O), sea level, atmospheric CO_2_, and global mean surface temperature, showing, as in Willeit et al., an excellent overall reproduction of the main features of the Plio–Pleistocene, including the long-term trend toward glaciation and cooling, and the occurrence of the MPT between 1.25 and 0.75 My BP. The fact that our continuous-execution results so closely resemble the time-splitting results of Willeit et al. strongly suggests that the long-term trends identified are robust and, as in Willeit et al., a consequence of the long-term forcing rather than, e.g., model drift.

One notable disagreement between model and proxy data occurs during Marine Isotope Stage 11 (MIS-11) interglacial between ~425,000 and 375,000 B.P. The model fails to reproduce the characteristics of this interglacial, including the substantial warming, ice loss, and sea level increase at this time. This is due to a known deficiency among models in capturing this unusually long interglacial (12). The comparison is further hampered, in our case, by the crude representation in CLIMBER-2 of the Greenland Ice Sheet, which could play a particularly important role in MIS-11.

During the first 1.5 My of the simulation (Late Pliocene and Early Pleistocene), the concentration of carbon dioxide in the atmosphere roughly exceeds 300 ppm, and simulated global surface air temperatures exceed (by as much as 1.4 °C) preindustrial levels. As we near the MPT, we observe a decrease in CO_2_ and associated cooling. Assisted by the process of regolith removal by ice flow over successive glaciations, ice volume accumulates, larger ice sheets take hold, and global temperatures decrease substantially. The cyclicity of glacial–interglacial cycles evolves, over the course of the MPT, from low-amplitude, roughly sinusoidal 20 ky and 40 ky precession and obliquity-driven responses to an increasing amplitude, strongly asymmetric ~100,000-y “sawtooth” response, despite any substantial changes in orbital forcing. Willeit et al. show that this behavior is associated with the dynamics of very large continental ice sheets favored by both the long-term volcanic outgassing and regolith removal from successive glacial advance and retreat cycles.

#### FIT vs. BIT experiments.

Having reproduced the basic results of Willeit et al., i.e., the main features of the glacial/interglacial cycles of the last 3 My including the MPT, we performed additional experiments to assess potential path dependence and hysteresis in the Plio–Pleistocene climate evolution. These experiments include “FIT” simulations similar to those performed by Willeit et al., and counterfactual “BIT” simulations where the model is run backward from the preindustrial present to 3 My BP. In practice, this is achieved by forward execution of the model, initializing the model with a preindustrial state and driving the model with time-reversed versions of the forcings (orbital parameters, CO_2_ volcanic outgassing, and regolith cover). These simulations provide insight into the consequences of nonlinearities and prior state dependence in the specific course of evolution of the climate system over the Plio–Pleistocene.

The reversal of the process of regolith removal in the time-reversed simulations is arguably unphysical (it would imply a decrease in entropy with time). It is still of scientific interest to perform the experiment, however, in assessing the path-dependence of the entire system. So we consider several alternative scenarios: one in which regolith removal a) indeed operates in reverse, and scenarios where regolith is kept constant in the reverse simulations, including b) at its modern depleted state (which is what the true initial condition would in fact be if time was suddenly run backward) and c) at its ancient nondepleted state (which is still instructive from the standpoint of the behavior of the system). This is detailed further in *Materials and Methods*.

**Fig. 2.**
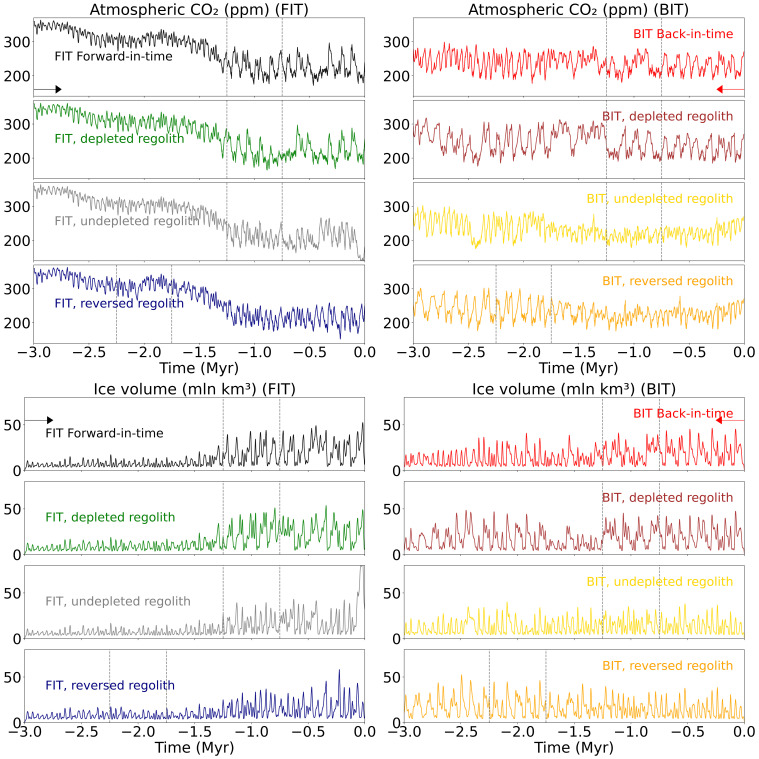
Atmospheric CO_2_ and ice volume in regolith removal experiments. To analyze the role of the regolith in the mid-Pleistocene transition, and in general, over the last 3 My, we performed 8 simulations: 4 FIT and 4 BIT (see the procedure detailed in *Materials and Methods*, summarized in *SI Appendix*, Fig. S1). The first FIT is with actual regolith, decreasing from −3 My to the present. Two other simulations with steady regolith as in the present (depleted) or in ancient times (undepleted) and finally reversing them. The equivalent BIT simulations have been run by applying the same forcings in the inverse direction in time. *Left* column: FIT executions. *Right* column, BIT executions by reversing all forcings. Black/red: FIT/BIT executions. Green/brown: FIT/BIT keeping the regolith constant as in modern preindustrial value (depleted). Gray/yellow: FIT/BIT keeping the regolith constant as in −3 My ancient (undepleted) value. Blue/orange: FIT/BIT reversing sediment. The first FIT simulation, with actual sediment removal, reproduces the proxy measurements of the glacial/interglacial cycles over the last 3 My. The arrows indicate the direction of execution of the FIT and BIT experiments. The dashed vertical lines mark the MPT (time-reversed for the reversed sediment experiments).

With the BIT simulations, we observe marked path dependence in the behavior of the coupled system, as illustrated in Fig. 2 by both CO_2_ and ice volume. The increase in the prescribed (see *SI Appendix*, Fig. S1 for time series of all forcings) volcanic outgassing BIT does not generate the high atmospheric CO_2_ levels and relatively ice-free conditions of the Late Pliocene seen in the FIT simulations of the same model (Fig. 2). Such hysteresis appears regardless of the sediment configuration used. Whether regolith is removed, remains in place, or follows a time-reversed trajectory, atmospheric CO_2_ concentrations remain low and ice volume high during the late Pliocene and early Pleistocene in the BIT simulations relative to the FIT simulations. While the FIT simulations start with atmospheric CO_2_ levels exceeding 360 ppm in the Late Pliocene, none of the four BIT experiments ever breach 300 ppm. We attribute this disparity to path dependence in carbon cycle dynamics, explored in detail in the following section.

**Fig. 3.**
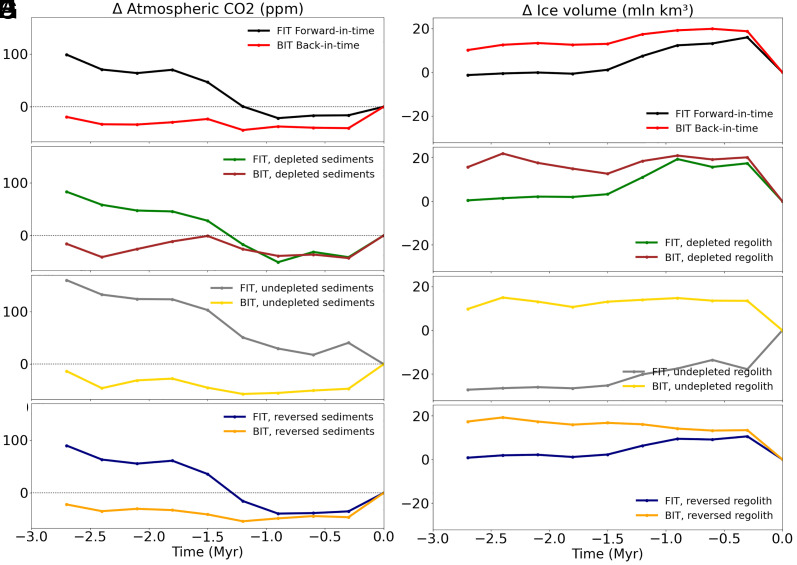
Atmospheric CO_2_ and ice volume anomalies as a function of time (My). Atmospheric CO_2_ concentration (ppm) (*Left*) and ice volume anomalies (mln km^3^) (*Right*) have been calculated with respect to the Present, averaging every 300 ky to analyze long-term trends and represented against time (My). (*A*, *B*) Black/red: FIT execution (black) with actual regolith, decreasing from −3 My to the present compared with BIT (red), same forcings but reversing the time. (*C*, *D*) Green/brown: FIT/BIT keeping the regolith constant as in modern preindustrial value (depleted). (*E, F*) Gray/yellow: FIT/BIT keeping the regolith constant as in −3 My ancient (undepleted) value. (*G*, *H*) Blue/orange: FIT/BIT reversing regolith.

To better illustrate the hysteresis properties, we have plotted the time-averaged (300 ky mean) results for the paired FIT and BIT simulations for each of the alternative regolith scenarios (Fig. 3) The results for FIT and BIT experiments diverge substantially prior to ~1.5 My BP, exceeding 100 ppm atmospheric CO_2_ and 10 M km^3^ in ice volume during the early Pliocene.

While this divergence is seen for each of the regolith scenarios, it is most pronounced for the constant, undepleted regolith. This is due to the fact that the FIT experiments in this case never encounter the regolith-free conditions that favor very large ice sheets, so global ice volume and ocean carbon storage remain low, and atmospheric CO_2_ levels high, over much of the simulation.

It should be noted that the plots shown in Fig. 3 are not hysteresis curves in the standard sense, since the FIT and BIT curves have not yet merged at the earliest times (3 My BP) of our simulations. A worthwhile extension of this work would involve extended simulations that reach further back in time through the Pliocene and into the late Miocene.

#### Path-dependent carbon cycle dynamics.

**Fig. 4.**
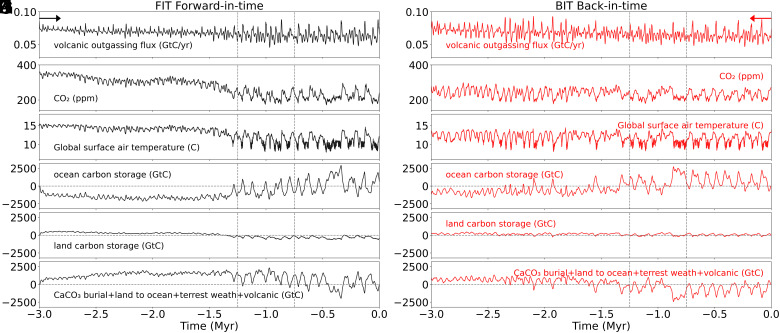
Dynamics of carbon cycle pools in experiments. *Left* column: FIT execution. *Right* column: BIT execution by reversing all forcings. (Panels *A* and *B*) Volcanic outgassing flux (GtC/yr), (*C* and *D*) atmospheric carbon dioxide concentration in ppm, (*E* and *F*) global surface air temperature (*C*), (*G* and *H*) total ocean carbon change (GtC), (*I* and *J*) total land carbon change (GtC), (*K* and *L*) sum of total carbon change in CaCO_3_ burial, flux land to the ocean, Terrestrial weathering, and volcanic outgassing (GtC). The arrows indicate the direction of execution of the FIT and BIT experiments. The dashed vertical lines mark the MPT.

The disparity in atmospheric CO_2_ arises from nonlinear behavior in the ocean (35) and terrestrial carbon cycle. Although both experiments have the same prescribed forcing, the increase back in time in volcanic outgassing, as noted earlier, does not, in the BIT experiments, generate the high CO_2_ levels of the Late Pliocene seen in the FIT experiments. Note that differences in volcanic outgassing history in the two experiments are due to ice volume changes, which impact outgassing as characterized in previous work (36, 37) and differ between the FIT and BIT Experiments (see ice volume in Fig. 2). While a more detailed analysis of the governing carbon cycle dynamics remains the topic of future investigation, we can understand this behavior in terms of the different carbon cycle responses in the two cases (Fig. 4). In the FIT simulations, the pre-MPT interval (first 1.5 My BP) shows an average of ~900 GtC less ocean carbon content than the BIT simulations. In the BIT simulations, the initial state is characterized by modern preindustrial conditions and considerably lower (~280 ppm) CO_2_ levels than the substantially higher (~350 ppm CO_2_) late Pliocene initial greenhouse state of the FIT simulations. The oceans remain cool and stably stratified, favoring substantial continued ocean carbon storage which mitigates the rise in atmospheric CO_2_ otherwise expected as volcanic outgassing increases back in time. Instead of a slow, steady trend toward a Pliocene-like greenhouse state back in time, we observe a persistent quasiglacial state of relatively low (less than 300 ppm) CO_2_ levels and (under 13 °C) global mean temperature. The increase in ocean carbon content is partly offset by decreased (~700 GtC) carbon burial from decreased weathering and CaCO_3_ ocean deposition due to cooler ocean surface temperatures and a weaker hydrological cycle. These two opposite sign effects don’t quite cancel, with a residual increase in net carbon burial of ~200 GtC, consistent with the roughly 100 ppm lower (~200 to 250 ppm vs. 300 to 350 ppm) average pre-MPT CO_2_ levels in the BIT experiments (see *SI Appendix*, Fig. S2 for details).

The greater solubility of carbon in cold glacial oceans leads to a lowering of atmospheric CO_2_ levels. Changes in the oceanic carbon sink are attributed to physical or biological mechanisms (35) such as changes in ocean temperatures and solubility, the volume of the Southern Ocean’s deep water masses (38), changes in marine biology (39) and ocean carbon pool imbalance associated with different time scales in bicarbonate land-to-ocean flux and CaCO_3_ burial flux. A more detailed exploration of the behavior of the ocean and terrestrial carbon cycle behavior in these simulations is currently being pursued and will be described in forthcoming work.

#### Mid-Pleistocene transition.

**Fig. 5.**
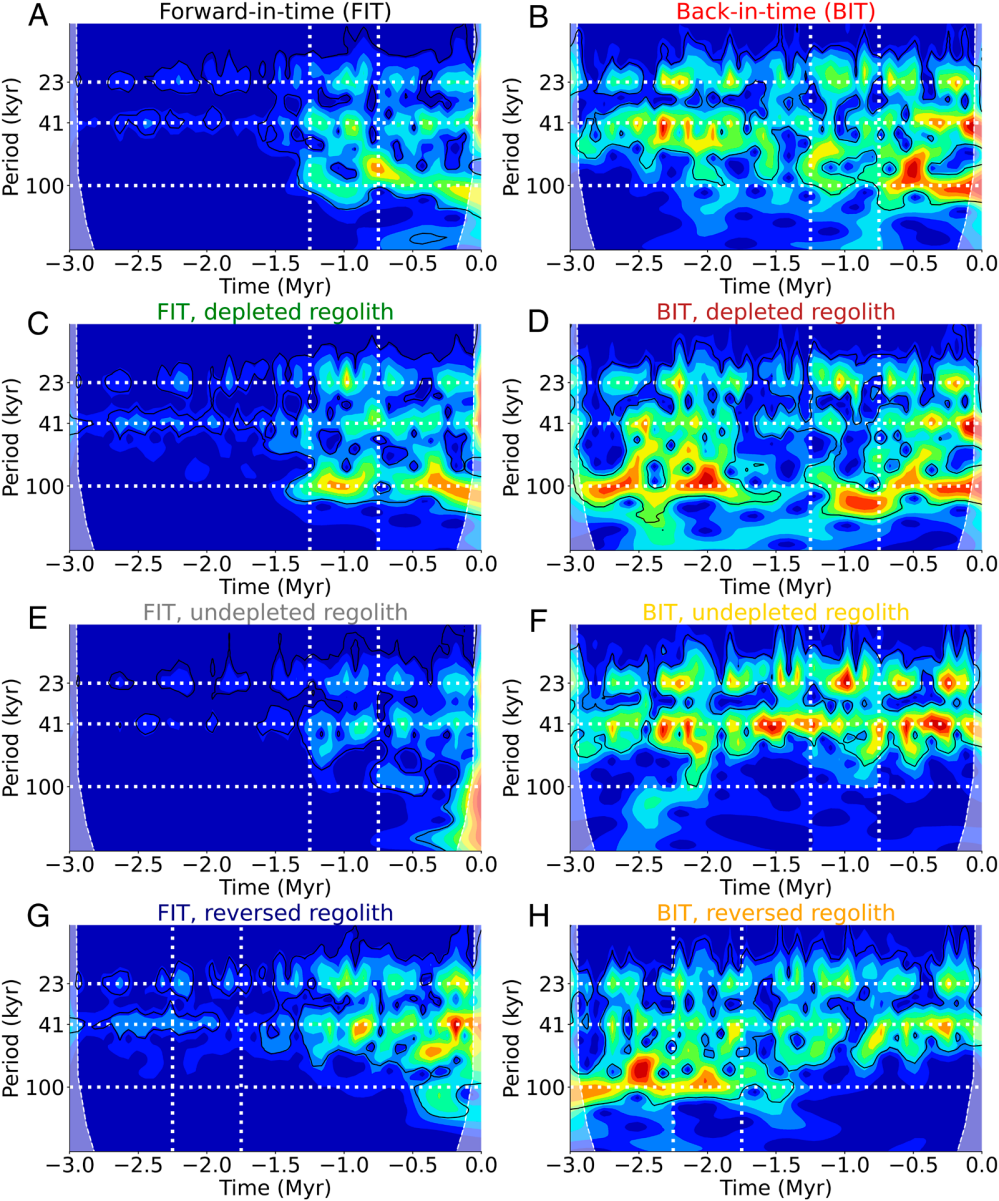
Wavelet (42) spectra of ice volume. Black contours indicate the 5% significance level against red noise. *Left* column: FIT executions. *Right* column: BIT executions by reversing all forcings. (*A*, *B*) Black/red: FIT/BIT executions; the black-one reproduces proxies. (*C*, *D*) Green/brown: FIT/BIT keeping the regolith constant as in modern preindustrial value (depleted). (*E*, *F*) Gray/yellow: FIT/BIT keeping the regolith constant as in −3 My ancient (undepleted) value. (*G*, *H*) Blue/orange: FIT/BIT reversing sediment. For each of the 8 simulations the normalized amplitude evaluated in 1,000-y steps is indicated by a color scale shown at the *Bottom* of the plot. The horizontal white lines represent the orbital periods of precession (∼23 ky), obliquity (∼41 ky), and eccentricity (∼100 ky). The dashed vertical lines mark the MPT (time-reversed for the reversed sediment experiments).

To assess the spectral characteristics of the climate evolution for the various scenarios, we perform a wavelet analysis on the glacial ice volume series (Fig. 5). For the standard FIT experiment (panel A), we see the well-established pattern (40, 41) of low-amplitude precession- and obliquity-driven 20 ky and 40 ky variability prior to the MPT, and larger-amplitude ~100 ky variability after the MPT, with the transition in character taking place within the MPT (−1.25 to −0.75 My). Essentially the same results are obtained (panel C) for the constant depleted sediment FIT experiment, suggesting that the CO_2_ history, rather than the precise evolution of sediment depletion, most fundamentally determines the onset of the MPT. The MPT is not observed, on the other hand, for either the constant undepleted (panel E) or reversed sediment (panel G) experiments, both of which lack spectral power at the 100 ky timescale throughout the duration of the simulation. Collectively, these results suggest that depleted regolith and lowered CO_2_ levels are required to produce the 100 ky sawtooth cycle.

Additional insights are derived from the wavelet analyses of the BIT experiments. The most striking observation is that precession and obliquity-driven variability is uniformly greater in amplitude throughout the simulated time interval (Fig. 5), consistent with the more persistent glacial conditions found in the BIT simulations. In the standard BIT experiment (panel B), we additionally observe the MPT to take place during the same time interval (−1.25 to −0.75 My) as in the corresponding FIT experiment. This is as expected since the same combination of low CO_2_ and depleted regolith is present at that time. However, as noted earlier, this is arguably an unphysical scenario since regolith cannot spontaneously reassemble back in time.

A more physically consistent scenario is that once the supply of regolith is depleted, it remains depleted. In this scenario, where we assume constant depleted sediment, the 100 ky variability is present uniformly throughout the full simulated interval (panel D). In this case, we have the required combination of factors (low CO_2_ and depleted regolith) present throughout the entire simulation. On the other hand, 100 ky variability is entirely absent in the constant undepleted sediment simulation (panel F), since only one of the two required factors (low CO_2_) is present. Finally, the MPT is observed instead during the mirrored time interval of −2.25 My to −1.75 My, and in the opposite direction (the 100 ky signal disappears rather than appears) in the reversed sediment experiment (panel H), as expected since the required factors (low CO_2_ and depleted regolith) are only present during the early part of the simulation.

These experiments are extremely clarifying with respect to the origins of the 100 ky signal. It is seen to be a robust and predictable consequence of the dynamics of large ice sheets favored by low CO_2_ combined with depleted regolith.

## Discussion

Using the CLIMBER-2 Earth System model, which includes the critical ocean, atmosphere, cryosphere, and carbon cycle processes and couplings needed for a comprehensive simulation of Plio–Pleistocene climate evolution, we investigated the evolution of Northern Hemisphere glaciation over the past 3 My. We find that this evolution is path dependent and, to be specific, not reversible in time. In experiments beginning with modern preindustrial conditions and driving the model back in time with time-reversed Earth orbital and tectonic forcing, the warm, relatively ice-free conditions of the late Pliocene and early Pleistocene are not reproduced. This hysteresis behavior is shown to be a consequence of nonlinearities in the ocean and terrestrial carbon cycle, including an initial state dependence wherein carbon storage by glacial oceans opposes the tendency for atmospheric CO_2_ increase derived from increased volcanic outgassing. Our experiments reinforce the picture of a Plio–Pleistocene climate system that allows for the coexistence of two regimes in the ocean–atmosphere–cryosphere carbon cycle system at the glacial/interglacial time scale: one with relatively high CO_2_, little ice, a persistent warm ocean, and small amplitude ~20 to 40 ky obliquity/precession-driven glacial/interglacial cycles, and the other with lower CO_2_, more extensive ice, a cold ocean, and high-amplitude ~100 ky glacial/interglacial cycles where eccentricity plays a greater role. These two attractors differ in the activation of feedbacks between ocean, ice, and carbon dynamics. The time interval of the past 3 My does not appear to capture a large enough range in external forcing to contain an entire hysteresis loop. Over the range of forcing analyzed, we thus see only transitions from the former to the latter regime.

This path-dependence also impacts the occurrence and timing of the so-called MPT from low-amplitude sinusoidal precession- (~23 ky) and obliquity- (~41 ky) driven glacial/interglacial cycles to high-amplitude ~100 ky sawtooth cycles seen between −1.25 My and −0.75 My, involving a much more nonlinear response to orbital forcing. While robustly present in our FIT experiments, we find that the MPT disappears in BIT integrations depending on how regolith depletion is handled. In the most physically realistic scenario, where regolith, once depleted in FIT integrations, remains so in time-reversed situations, the ~100 ky sawtooth cycle is uniformly present throughout the entire course of the time-reversed simulations. If regolith instead is kept in its undepleted state, there is no ~100 ky sawtooth cycle over the course of the time-reversed simulations. The 100 ky signal is consequently seen, in our experiment, to be a robust and predictable consequence of the dynamics of large ice sheets favored by low CO_2_ combined with depleted regolith. Further work will be necessary to confirm these findings and to better understand the key coupled ocean–atmosphere–biogeochemical mechanisms that underlie them.

## Materials and Methods

### Model and Experimental Design.

The CLIMBER-2 model (30, 36, 39, 43–45) is a global Earth system model of intermediate complexity which combines reasonable accuracy with computation speed, allowing simulations of hundreds of thousands of years at a time. It includes a 2.5-dimensional statistical-dynamical atmospheric model, a 2‐dimensional 3-basin ocean model, the 3-dimensional thermomechanical ice-sheet model SICOPOLIS applied only to the Northern Hemisphere, dynamical terrestrial vegetation model, ocean carbon cycle model, aeolian dust cycle, and other components. The ice sheet model is only applied to the Northern Hemisphere; the Antarctic ice sheet is assumed to contribute an additional 10% to global ice volume variation. The ocean carbon cycle includes modules for marine biota, oceanic biogeochemistry, and marine sediments. CLIMBER-2 works with a coarse spatial resolution, allowing only continental-scale features and different oceanic basins to be resolved. However, this parameterization allows a reasonable computational speed that completes simulations of 3 My in just over a month (24).

The carbon-cycle model (39) has been configured in an open C-cycle. Atmospheric CO_2_ concentration is computed by the model from the following equation, using a conversion factor of 0.47ppm GtC^−1^ (46).

[1] $$\begin{array}{c} \mathrm{Atmospheric}\;\mathrm{CO2}\;\mathrm{(ppm)}=\\ \mathrm{initial}\;\mathrm{CO2}\;\mathrm{prescribed}\;\mathrm{(ppm)}-0.47*\int_{0}^{t}\mathrm{(ocean}\;\mathrm{carbon+land}\;\mathrm{carbon+}\mathrm{CaCO3}\;\mathrm{burial+}\\ \mathrm{atmospheric}\;\mathrm{consumption}\;\mathrm{terrestrial}\;\mathrm{weathering+bicarbonate}\;\mathrm{flux}\;\mathrm{from}\;\mathrm{land}\;\mathrm{to}\;\mathrm{ocean+volcanic}\;\mathrm{out}\;\mathrm{gassing)dt}. \end{array}$$

CLIMBER-2 was run over the mid-Pliocene to Present, from 3 to 0 My BP, driven by prescribed gradual decrease in volcanic outgassing and regolith removal from basal ice interactions. The configuration was similar to that performed by Willeit et al. (23), but unlike this previous study, we did not use the time-splitting technique in this work. To analyze long-term processes related to the carbon cycle, our experiments were performed in a single run, leaving the model to evolve over 3 My. *SI Appendix* details the simulations carried out with different histories in the gradual regolith removal, selecting those that best reproduced the MPT vs. the proxy measurements, such as the reference FIT experiment.

Once the forcings configuration that best reproduce the glacial/interglacial cycles of the last 3 My was determined (FIT—black), with particular attention to the MPT, the BIT experiment (BIT—red) was designed by reversing all forcings: Solar cycle (eccentricity, obliquity, and longitude of perihelion), prescribed volcanic outgassing (23, 36), and sediment masks. After the simulation was run, the time axis was flipped.

To analyze the role of regolith, three additional experiments were performed forward and BIT: keeping them constant as in modern preindustrial value (depleted) (FIT—green/BIT—brown), keeping the regolith steady as in −3 My ancient (undepleted) value (FIT—Gray/BIT—Yellow), and finally reversing then (FIT—Blue/BIT—Orange). *SI Appendix*, Fig. S1 details the forcings prescribed in every experiment.

## Supplementary Material

Appendix 01 (PDF)

## Data Availability

All study data are included in the article and/or *SI Appendix*.

## References

[1] M. E. Raymo, D. W. Oppo, W. Curry, The Mid-Pleistocene climate transition: A deep sea carbon isotopic perspective. Paleoceanography **12**, 546–559 (1997).

[2] A. Abe-Ouchi , Insolation-driven 100,000-year glacial cycles and hysteresis of ice-sheet volume. Nature **500**, 190–193 (2013). 23925242 10.1038/nature12374

[3] B. Saltzman, Dynamical Paleoclimatology: Generalized Theory of Global Climate Change (Academic Press, 2002).

[4] K. Maasch, Statistical detection of the mid-Pleistocene transition. Clim. Dyn. **2**, 133–143 (1988).

[5] R. François , Contribution of Southern Ocean surface-water stratification to low atmospheric CO2 concentrations during the last glacial period. Nature **389**, 929–935 (1997).

[6] P. U. Clark , The middle Pleistocene transition: Characteristics, mechanisms, and implications for long-term changes in atmospheric pCO2. Q. Sci. Rev. **25**, 3150–3184 (2006).

[7] A. Berger, X. S. Li, M. F. Loutre, Modelling northern hemisphere ice volume over the last 3 Ma. Q. Sci. Rev. **18**, 1–11 (1999).

[8] J. D. Hays, J. Imbrie, N. J. Shackleton, Variations in the Earth’s orbit: Pacemaker of the ice ages. Science **194**, 1121–1132 (1976). 17790893 10.1126/science.194.4270.1121

[9] J. F. McManus, R. Francois, J.-M. Gherardi, L. D. Keigwin, S. Brown-Leger, Collapse and rapid resumption of Atlantic meridional circulation linked to deglacial climate changes. Nature **428**, 834–837 (2004). 15103371 10.1038/nature02494

[10] M. Mudelsee, M. Schulz, The Mid-Pleistocene climate transition: Onset of 100 ka cycle lags ice volume build-up by 280 ka. Earth Planet. Sci. Lett. **151**, 117–123 (1997).

[11] D. Paillard, The timing of Pleistocene glaciations from a simple multiple-state climate model. Nature **391**, 378–381 (1998).

[12] N. J. Shackleton, The 100,000-year ice-age cycle identified and found to lag temperature, carbon dioxide, and orbital eccentricity. Science **289**, 1897–1902 (2000). 10988063 10.1126/science.289.5486.1897

[13] T. B. Chalk , Causes of ice age intensification across the Mid-Pleistocene Transition. Proc. Natl. Acad. Sci. U.S.A. **114**, 13114–13119 (2017). 29180424 10.1073/pnas.1702143114 PMC5740680

[14] J. Imbrie , On the structure and origin of major glaciation cycles 1. Linear responses to Milankovitch forcing. Paleoceanography **7**, 701–738 (1992).

[15] J. Imbrie , On the structure and origin of major glaciation cycles 2. The 100,000-year cycle. Paleoceanography **8**, 699–735 (1993).

[16] N. G. Pisias, T. C. Moore, The evolution of Pleistocene climate: A time series approach. Earth Planet. Sci. Lett. **52**, 450–458 (1981).

[17] A. Ganopolski, Toward generalized Milankovitch theory (GMT). Clim. Past **20**, 151–185 (2024).

[18] S. Rutherford, S. D’Hondt, Early onset and tropical forcing of 100,000-year Pleistocene glacial cycles. Nature **408**, 72–75 (2000). 11081508 10.1038/35040533

[19] M. Y. Verbitsky, M. Crucifix, D. M. Volobuev, A theory of Pleistocene glacial rhythmicity. Earth Syst. Dyn. **9**, 1025–1043 (2018).

[20] T. Mitsui, M. Willeit, N. Boers, Synchronization phenomena observed in glacial–interglacial cycles simulated in an Earth system model of intermediate complexity. Earth Syst. Dyn. **14**, 1277–1294 (2023).

[21] R. Bintanja, R. S. W. van de Wal, North American ice-sheet dynamics and the onset of 100,000-year glacial cycles. Nature **454**, 869–872 (2008). 18704083 10.1038/nature07158

[22] P. U. Clark, D. Pollard, Origin of the Middle Pleistocene Transition by ice sheet erosion of regolith. Paleoceanography **13**, 1–9 (1998).

[23] M. Willeit, A. Ganopolski, R. Calov, V. Brovkin, Mid-Pleistocene transition in glacial cycles explained by declining CO2 and regolith removal. Sci. Adv. **5**, eaav7337 (2019). 30949580 10.1126/sciadv.aav7337 PMC6447376

[24] A. Ganopolski, R. Calov, The role of orbital forcing, carbon dioxide and regolith in 100 kyr glacial cycles. Clim. Past **7**, 1415–1425 (2011).

[25] A. Robinson, R. Calov, A. Ganopolski, Multistability and critical thresholds of the Greenland ice sheet. Nat. Clim. Change **2**, 429–432 (2012).

[26] J. Garbe, T. Albrecht, A. Levermann, J. F. Donges, R. Winkelmann, The hysteresis of the Antarctic Ice Sheet. Nature **585**, 538–544 (2020). 32968257 10.1038/s41586-020-2727-5

[27] A. Ganopolski, S. Rahmstorf, Rapid changes of glacial climate simulated in a coupled climate model. Nature **409**, 153–158 (2001). 11196631 10.1038/35051500

[28] T. L. Frölicher, F. Joos, Reversible and irreversible impacts of greenhouse gas emissions in multi-century projections with the NCAR global coupled carbon cycle-climate model. Clim. Dyn. **35**, 1439–1459 (2010).

[29] S.-W. Park, J.-S. Kug, A decline in atmospheric CO2 levels under negative emissions may enhance carbon retention in the terrestrial biosphere. Commun. Earth Environ. **3**, 1–8 (2022).

[30] A. Ganopolski , CLIMBER-2: A climate system model of intermediate complexity. Part II: Model sensitivity. Clim. Dyn. **17**, 735–751 (2001).

[31] L. E. Lisiecki, M. E. Raymo, A Pliocene-Pleistocene stack of 57 globally distributed benthic δ18O records. Paleoceanography **20**, PA1003 (2005).

[32] R. M. Spratt, L. E. Lisiecki, A Late Pleistocene sea level stack. Clim. Past **12**, 1079–1092 (2016).

[33] B. Bereiter , Revision of the EPICA Dome C CO2 record from 800 to 600 kyr before present. Geophys. Res. Lett. **42**, 542–549 (2015).

[34] C. W. Snyder, Evolution of global temperature over the past two million years. Nature **538**, 226–228 (2016). 27669024 10.1038/nature19798

[35] S. Rahmstorf, Ocean circulation and climate during the past 120,000 years. Nature **419**, 207–214 (2002). 12226675 10.1038/nature01090

[36] A. Ganopolski, V. Brovkin, Simulation of climate, ice sheets and CO_2_ evolution during the last four glacial cycles with an Earth system model of intermediate complexity. Clim. Past **13**, 1695–1716 (2017).

[37] P. Huybers, C. Langmuir, Feedback between deglaciation, volcanism, and atmospheric CO2. Earth Planet. Sci. Lett. **286**, 479–491 (2009).

[38] G. H. Denton , The last glacial termination. Science **328**, 1652–1656 (2010). 20576882 10.1126/science.1184119

[39] V. Brovkin, A. Ganopolski, D. Archer, G. Munhoven, Glacial CO_2_ cycle as a succession of key physical and biogeochemical processes. Clim. Past **8**, 251–264 (2012).

[40] M. E. Raymo, The timing of major climate terminations. Paleoceanography **12**, 577–585 (1997).

[41] J. Beer, W. Mende, R. Stellmacher, The role of the sun in climate forcing. Q. Sci. Rev. **19**, 403–415 (2000).

[42] D. Khider , Pyleoclim: Paleoclimate timeseries analysis and visualization with python. Paleoceanogr. Paleoclimatol. **37**, e2022PA004509 (2022).

[43] V. Brovkin, A. Ganopolski, D. Archer, S. Rahmstorf, Lowering of glacial atmospheric CO2 in response to changes in oceanic circulation and marine biogeochemistry. Paleoceanography **22**, PA4202 (2007).

[44] V. Petoukhov , CLIMBER-2: A climate system model of intermediate complexity. Part I: Model description and performance for present climate. Clim. Dyn. **16**, 1–17 (2000).

[45] V. Brovkin , Carbon cycle, vegetation, and climate dynamics in the Holocene: Experiments with the CLIMBER-2 model. Glob. Biogeochem. Cycles **16**, 86-1–86-20 (2002).

[46] K. L. Denman , “Couplings Between Changes in the Climate System and Biogeochemistry” in The Physical Science Basis, Contribution of Working Group I to the Fourth Assessment Report of the Intergovernmental Panel on Climate Change, S. Solomon , Eds. (Cambridge University Press, Cambridge, 2007), pp. 501–587.

