# Supplementary material for: Path-dependence of the Plio–Pleistocene glacial/interglacial cycles
Source: Proc Natl Acad Sci U S A. 2024 Jun 17;121(26):e2322926121. doi: 10.1073/pnas.2322926121 (PMC11214093; doi:10.1073/pnas.2322926121)
Supplement: Supplementary file 1 — Appendix 01 (PDF) [file pnas.2322926121.sapp.pdf]

Supporting Information for
Path-Dependence of the Plio-Pleistocene Glacial/Interglacial Cycles

Judit Carrillo*[1], Michael E. Mann*[1], Christopher J. Larson[1], Shannon Christiansen[1], Matteo Willeit[2], Andrey Ganopolski[2], Xueke Li[1] and Jack G. Murphy[1]

(1) University of Pennsylvania, Department of Earth and Environmental Science, Philadelphia, United States
(2) Potsdam Institute for Climate Impact Research, Potsdam, Germany

*To whom correspondence may be addressed.
Email: juditcp@sas.upenn.edu, mmann00@sas.upenn.edu

This PDF file includes:

Figures S1 to S2
SI Reference

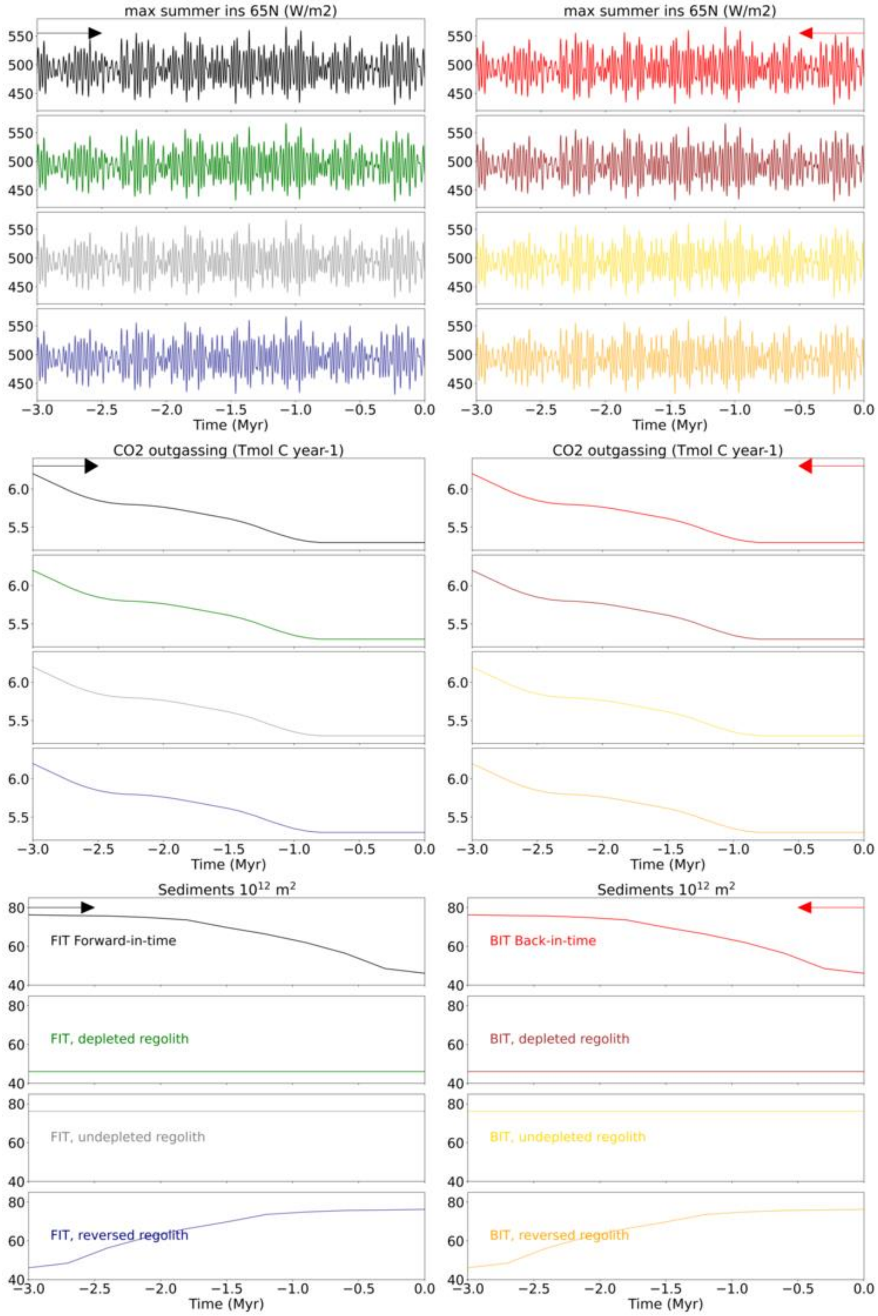

Fig. S1. Description of the experiments. Forcings prescribed in the experiments: Maximum summer insolation in 65ºN (W/m^2), CO2 volcanic outgassing (Tmol C $year^{-1}$), and mask of regolith[1] (10^{12} m^2). Left column: Forward-in-time executions (FIT). Right column: Back-in-time executions by reversing all forcings (BIT). Black/Red: FIT/BIT experiments. Green/Brown: FIT/BIT keeping the regolith constant as in modern pre-industrial value (depleted). Gray/Yellow: FIT/BIT keeping the regolith constant as in -3 Myr ancient (undepleted) value. Blue/Orange: FIT/BIT reversing regolith. The arrows indicate the direction of execution of the FIT and BIT experiments.

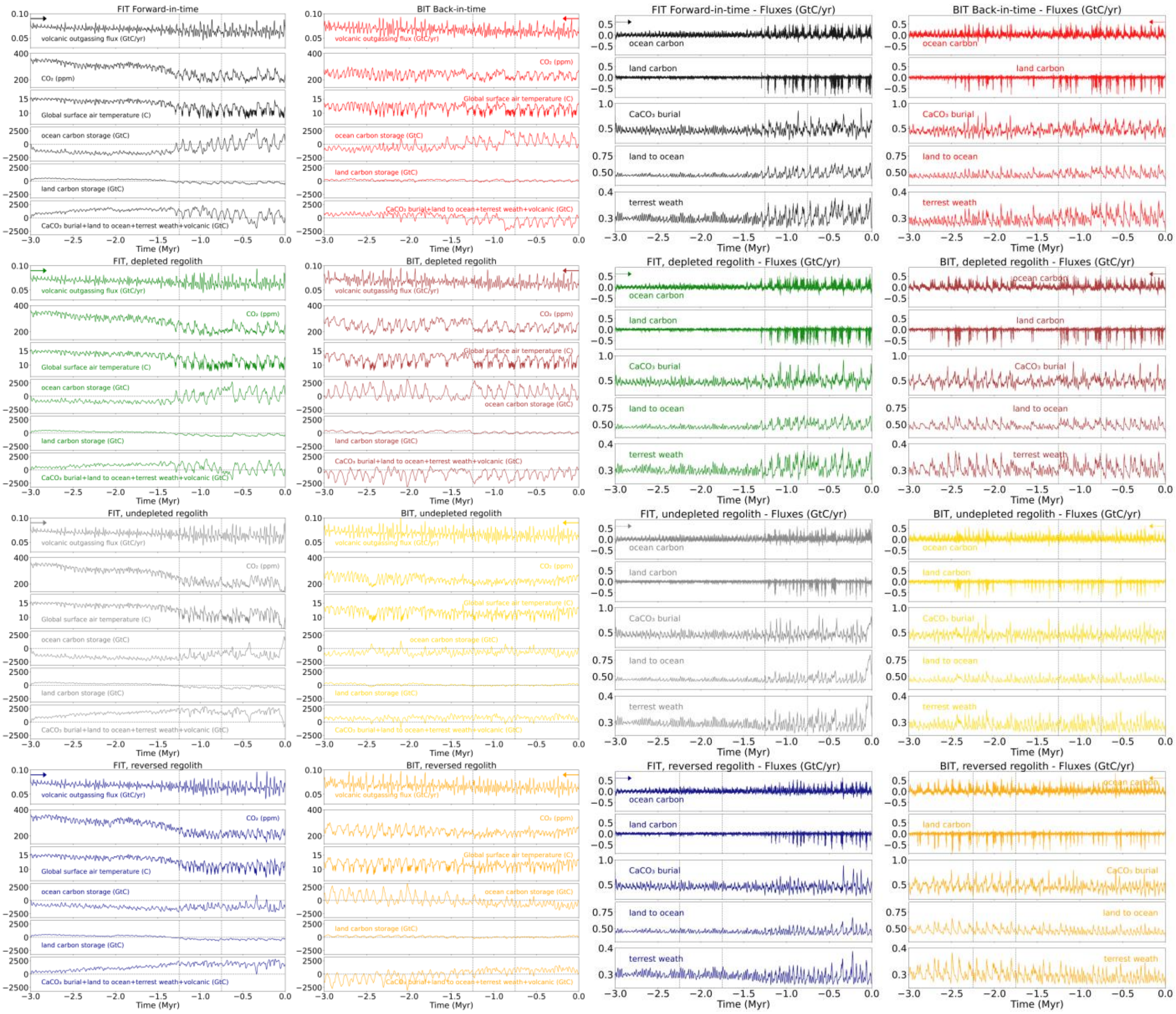

Fig. S2. Dynamics of Carbon cycle Pools and fluxes in regolith removal experiments. Left columns: (1st row) Volcanic outgassing flux (GtC/yr), (2nd row) Atmospheric carbon dioxide concentration in ppm, (3rd row) Global surface air temperature (C), (4th row) Total ocean carbon change (GtC), (5th row) Total land carbon change (GtC), (6th row) Sum of total carbon change in CaCO3 burial, Flux land to the ocean, Terrestrial weathering, and volcanic outgassing (GtC). Right columns: Carbon cycle fluxes (GtC/yr): Ocean carbon flux, Land carbon flux, CaCO3 burial, Flux land to the ocean, and Terrestrial weathering. Experiments: Black/Red: Forward-in-time (FIT)/ Back-in-time (BIT) executions. Green/Brown: FIT/BIT keeping the regolith constant as in modern pre-industrial value (depleted). Gray/Yellow: FIT/BIT keeping the regolith stable as in -3 Myr ancient (undepleted) value. Blue/Orange: FIT/BIT reversing regolith. The arrows indicate the direction of execution of the FIT and BIT experiments. The dashed vertical lines mark the MPT (time-reversed for the reversed regolith experiments).

Reference:

1. Willeit, M., Ganopolski, A., Calov, R. & Brovkin, V. Mid-Pleistocene transition in glacial cycles explained by declining CO2 and regolith removal. *Sci. Adv.* **5**, eaav7337 (2019).
